# Management of nausea and vomiting induced by antibody–drug conjugates

**DOI:** 10.1007/s12282-025-01670-1

**Published:** 2025-01-29

**Authors:** Jawhara Farhat, Hitomi Sakai, Junji Tsurutani

**Affiliations:** 1 Université Claude Bernard Lyon 1, Lyon, France; 2 Advanced Cancer Translational Research Institute, Showa University, 1-5-8 Hatanodai, Shinagawa-ku, Tokyo, 142-8555 Japan

**Keywords:** Antibody–drug conjugate, Nausea and vomiting, Trastuzumab deruxtecan, Sacituzumab govitecan, Datopotamab deruxtecan

## Abstract

Antibody–drug conjugates (ADCs) are an emerging class of anticancer therapy that combines the specificity and long circulation half-life of monoclonal antibodies with the cytotoxic potency of the payload connected through a chemical linker. The optimal management of toxicities is crucial for improving quality of life in patients undergoing ADCs and for avoiding improper dose reductions or discontinuations. This article focuses on the characteristics and management of nausea and vomiting (NV) induced by three ADCs: trastuzumab deruxtecan (T-DXd), sacituzumab govitecan (SG), and datopotamab deruxtecan (Dato-DXd). We summarize the proposed mechanism of NV, clinical study data on NV, and recommendations from clinical guidelines. We also discuss three prospective studies evaluating prophylactic antiemetic therapy in patients receiving T-DXd, along with future perspectives.

## Introduction

Antibody‒drug conjugates (ADCs) have revolutionized cancer treatment by providing a more targeted approach to delivering chemotherapy. ADCs are designed to combine the specificity and long circulation half-life of monoclonal antibodies with the cytotoxic potency of the payload connected through a chemical linker [1]. The antibody in the ADC specifically binds to a target antigen on the surface of cancer cells, allowing the attached cytotoxic drug to be delivered directly to the tumor [1]. This precision minimizes the exposure of healthy cells to toxic agents, thereby reducing off-target side effects; however, patients receiving ADC treatment experience various side effects [2–5].

Nausea and vomiting (NV) are common side effects associated with trastuzumab deruxtecan (T-DXd), sacituzumab govitecan (SG), and datopotamab deruxtecan (Dato-DXd) [2–5]. Clinicians need to understand the symptom characteristics of NV induced by these ADCs and manage these symptoms effectively because NV can induce medical complications and distress and impair the quality of life of patients [6].

The purpose of this narrative review is to summarize the characteristics of NV induced by these ADCs and to outline strategies for their prevention and management.

## Pathophysiology of CINV

**Fig. 1:**
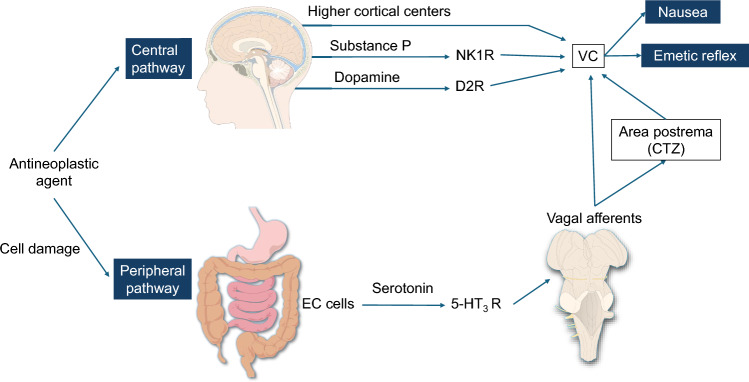
Central and peripheral mechanisms of CINV. The pathways by which antineoplastic agents, including antibody–drug conjugates, may produce an emetic response are shown. *VC* vomiting center, *CTZ* chemoreceptor trigger zone, *EC cells* enterochromaffin cells, *NK1R* neurokinin 1 receptor, *D2R* dopamine D2 receptor, *5-HT3 R* serotonin type 3 receptor

Although the mechanism of NV induced by ADCs has not been fully described, cytotoxic payloads are suspected to cause NV in a manner similar to that of cytotoxic chemotherapeutic drugs (Fig. 1).

The emetic response involves multiple organs and neurotransmitters, with two main pathways playing key roles: a peripheral pathway and a central pathway [7, 8]. The peripheral pathway, which is predominantly involved in the acute phase of emesis, begins with the release of serotonin from enterochromaffin cells in the gastrointestinal tract in response to chemotherapy-induced damage. Serotonin activates 5-hydroxytryptamine type 3 receptors (5-HT3 R) on vagal afferent nerves, which transmit the stimulus to the nucleus tractus solitarius (NTS) and dorsal motor nucleus of the vagus (DMNV), triggering the emetic response [7–9]. The central pathway is predominantly involved in the delayed phase of emesis. The chemoreceptor trigger zone (CTZ), also known as the area postrema, is located in the medulla oblongata at the base of the fourth ventricle, outside the blood‒brain barrier. This location allows it to be more susceptible to circulating substances, such as cytotoxic agents. When the CTZ is activated, it releases neurotransmitters such as serotonin, dopamine, and substance P. These neurotransmitters then stimulate the NTS and the DMNV through serotonin, dopamine, and neurokinin 1 (NK1) receptors [7, 8]. In addition, other pathways, such as the limbic system, vestibular system, and cerebral cortex, can stimulate emesis under conditions such as pain, depression, vertigo, motion sickness, or anticipatory NV. However, the neurotransmitters involved in these pathways are less well understood [10].

The mechanism of nausea is more complex than that of vomiting. The sensation of nausea is thought to arise from the activation of cortical structures involved in conscious perception [9]. Controlling nausea is usually more difficult than controlling vomiting, even when treatment with an NK1 receptor antagonist (NK1RA) or 5-HT3 receptor antagonist (5-HT3 RA) is administered [9, 11].

NV caused by T-DXd can persist for more than 120 h. However, it remains unclear whether this prolonged NV is solely due to the drug’s long half-life (approximately 6 days) or if other factors are involved [12]. T-DXd has demonstrated efficacy in treating brain metastases, suggesting that it penetrates the blood–brain barrier [13]. The potential direct effects of intracranial T-DXd on NV are yet to be fully understood, and it is anticipated that further investigations through basic and translational research will shed light on these mechanisms.

## Risk factors for NV

The risk of NV during chemotherapy varies with clinical factors, including the drug type and host-related variables. Chemotherapies are categorized into four risk groups according to the incidence of emesis without the use of antiemetic prophylaxis: high, moderate, low, and minimal risk [14–17]. Vomiting, rather than nausea, is used as the indicator for the appropriate category.

Previously reported risk factors for chemotherapy-induced nausea and vomiting (CINV) include younger age, female sex, a history of CINV, motion sickness, morning sickness during pregnancy, little or no alcohol use, nonsmoking, and anxiety and/or high pretreatment expectations of nausea and/or vomiting [18, 19]. There is no data to determine whether these are risk factors for ADC-induced NV.

## Trastuzumab deruxtecan

T-DXd is an ADC consisting of a humanized, anti-HER2 IgG1 monoclonal antibody that is covalently linked to a topoisomerase I inhibitor payload via a cleavable tetrapeptide-based linker. The drug-to-antibody ratio (DAR) of T-DXd is 8. This high DAR allows delivery of a high concentration of the cytotoxic payload DXd to the target cells [20].

Approximately 70% of patients receiving T-DXd treatment were found to experience nausea of all grades, and approximately 40% experienced vomiting of all grades in DESTINY clinical studies (Table 1) [21]. Recent clinical trials have shown only a slight decrease in frequency. The incidence of NV is highest in the first cycle and decreases in subsequent cycles. Most patients experienced grade 1 or 2 NV. Grade 3 nausea occurred in 5.8%, Grade 3 vomiting occurred in 2.4%, and Grade 4 vomiting occurred in 0.2% of the patients who received T-DXd according to the pooled analysis of the DESTINY clinical studies [21]. In the DESTINY-Breast04 and DESTINY-Breast06 studies, the time to definitive deterioration of the NV subscale was consistently shorter in the T-DXd group than in the control group [22, 23]. Although the protocols of DESTINY clinical studies have recommended the use of prophylactic antiemetic therapy with at least two agents for T-DXd treatment since 2020, this is not mandatory [2, 3].

**Table 1:** Incidence and severity of NV among patients receiving T-DXd in clinical studies

Study name Phase	Cancer type	*N*^a^	Dose and schedule of T-DXd	Nausea (%)	Vomiting (%)
DESTINY-Breast01 [27] Phase II	HER2-positive metastatic breast cancer	184	5.4 mg/kg, day 1 triweekly	Any grade: 78 Grade 3: 8 Following grades: 0	Any grade: 46 Grade 3: 4 Following grades: 0
DESTINY-Breast02 [28] Phase III	HER2-positive metastatic breast cancer	404	5.4 mg/kg, day 1 triweekly	Any grade: 73 Grade 3: 7	Any grade: 38 Grade ≥ 3: 4
DESTINY-Breast03 [2] Phase III	HER2-positive metastatic breast cancer	257	5.4 mg/kg, day 1 triweekly	Any grade: 73 Grade ≥ 3: 7	Any grade: 44 Grade ≥ 3: 2
DESTINY-Breast04 [3] Phase III	HER2-low metastatic breast cancer	371	5.4 mg/kg, day 1 triweekly	Any grade: 73 Grade ≥ 3: 5	Any grade: 34 Grade ≥ 3: 1
DESTINY-Lung01 [29] Phase II	HER2-mutant non-small cell lung cancer	91	6.4 mg/kg, day 1 triweekly	Any grade: 73 Grade 3: 9 Following grades: 0	Any grade: 40 Grade 3: 3 Following grades: 0
DESTINY-Lung02 [30] Phase II	HER2-mutant non-small cell lung cancer	101	5.4 mg/kg, day 1 triweekly	Any grade: 67 Grade ≥ 3: 4	Any grade: 32 Grade ≥ 3: 3
		50	6.4 mg/kg, day 1 triweekly	Any grade: 82 Grade ≥ 3: 6	Any grade: 44 Grade ≥ 3: 2
DESTINY-Gastric01 [31] Phase II	HER2-positive gastric cancer	125	6.4 mg/kg, day 1 triweekly	Any grade: 63 Grade 3: 5 Following grades: 0	Any grade: 26 Grade ≥ 3: 0
DESTINY-Gastric02 [32] Phase II	HER2-positive gastric cancer	79	6.4 mg/kg, day 1 triweekly	Any grade: 67 Grade3: 8 Following grades: 0	Any grade: 44 Grade3: 3 Following grades: 0
DESTINY-PanTumor02 [33] Phase II	HER2-expressing solid tumors	267	5.4 mg/kg, day 1 triweekly	Any grade: 55	Any grade: 25

^a^ Number of patients who received T-DXd and were evaluated for safety

The emetogenic risk of T-DXd and the recommended antiemetic therapy are inconsistent across clinical guidelines. The MASCC and ESMO guidelines classify T-DXd at the high end of the moderate-risk category, similar to carboplatin [15]. The National Comprehensive Cancer Network (NCCN) guidelines recently upgraded the emetogenic risk of T-DXd from moderate to high [14]. This reclassification was based on the clinical experience of NCCN panel members and retrospective institutional data showing that some patients, who initially received standard prophylaxis for moderately emetogenic treatments, required the addition of an NK1 receptor antagonist in subsequent cycles owing to poor CINV control [14]. In other words, this reclassification is not based on data from prospective studies or large cohort studies. ASCO continues to classify T-DXd as moderate risk [16]. The Japan Society of Clinical Oncology (JSCO) guidelines classify T-DXd as the high end of the moderate-risk category. Because the classification of emetogenic risk varies across guidelines, the recommended antiemetic therapies range from two-drug regimens to four-drug regimens (Table 2).

**Table 2:** Emetic risk and recommended antiemetic therapy in recent guidelines

Guidelines	Last updated	Emetic risk of T-DXd	Recommended antiemetic therapy
MASCC and ESMO [15]	2023	High end of the moderate-risk category, most closely resembling that of carboplatin	NK_1_-RA, 5-HT3 RA, and DEX^a^
NCCN [14]	2024	High risk^b^	(A) NK_1_-RA, 5-HT3 RA, DEX, and OLZ (B) 5-HT3 RA, DEX, and OLZ (C) NK_1_-RA, 5-HT3 RA, DEX
ASCO [16]	2020	Moderate risk	5-HT3 RA and DEX
JSCO [17]	2023	High end of the moderate-risk category	5-HT3 RA and DEX^c^

^a^ The recommended regimen for carboplatin AUC ≥ 5

^b^ T-DXd was previously categorized as moderately emetogenic on the basis of clinical trial data. However, it was recategorized as highly emetogenic in the NCCN Guidelines owing to the clinical experience of panel members with this agent and institution-specific retrospective data

^c^ The guidelines state that there is potential for T-DXd to be considered a candidate for combination with NK1-RA-based triplet therapy

*MASCC* Multinational Association of Supportive Care in Cancer, *ESMO* European Society for Medical Oncology, *NCCN* National Comprehensive Cancer Network, *JSCO* Japan Society of Clinical Oncology, *T-DXd* trastuzumab deruxtecan, *NK1-RA* neurokinin 1 receptor antagonist, *5-HT3 RA* serotonin type 3 receptor antagonist, *DEX *dexamethasone

Three prospective clinical studies evaluated antiemetic therapy for patients receiving T-DXd treatment (Table 3). The EN-hance study was a multicenter randomized open-label study in patients with HER2-positive gastric cancer undergoing the first cycle of T-DXd [24]. The dose of T-DXd used was 6.4 mg/kg, which was administered intravenously on day 1 of a triweekly cycle; this dose differs from the standard dose of 5.4 mg/kg used for other cancers. The patients (n = 60) were randomized to a doublet regimen (dexamethasone and palonosetron) or a triplet regimen (aprepitant in addition to the doublet regimen). The complete response rate, defined as no vomiting and no rescue medication use, during the overall period (days 1–21) was 37.9% in the triplet regimen group and 41.4% in the doublet regimen group, which did not meet the prespecified antiemetic CR rate (≥ 18 of 29 patients). The median time for the first emetic event or nausea was approximately 3 days, with no difference between the two regimens. Iihara et al. conducted a multicenter randomized open-label exploratory study in patients with breast cancer receiving T-DXd [25]. The patients (*n* = 40) were randomized to a doublet regimen (dexamethasone and granisetron) or a triplet regimen (aprepitant in addition to the doublet regimen). The CR rates during the overall phase (0–120 h) were 36.8% in the doublet regimen group and 70.0% in the triplet regimen group (odds ratio [OR]: 0.1334; 95% confidence interval [CI] 0.0232–0.7672; *p* = 0.0190), with a difference of 33.2%. The CR rates during the extended-overall phase (0–168 h) were 31.6% in the doublet regimen group and 70.0% in the triplet regimen group (OR: 0.1073; 95% CI 0.0185–0.6239; *p* = 0.0087), with a difference of 38.4%. These two studies, which compared the efficacy of adding an NK1RA to 5-HT3 RA and dexamethasone in different populations with relatively small sample sizes, yielded inconsistent results. Notably, both studies were open-label and utilized patient-reported outcomes to assess NV. The ERICA study by Sakai et al. was a multicenter randomized, double-blind, placebo-controlled study in patients with HER2-positive or HER2-low breast cancer who were undergoing the first cycle of T-DXd [26]. This study focused on persistent NV induced by T-DXd treatment and utilized patient-reported outcomes to assess NV. Patients (*n* = 168) were randomized to olanzapine 5 mg or placebo once daily from days 1–6 in addition to 5-HT3 RA and dexamethasone (6.6 mg intravenously or 8 mg orally on day 1). The CR rate in the delayed phase (24–120 h) was 70.0% in the olanzapine group and 56.1% in the placebo group, with a difference of 13.9% and a two-sided 60% CI of 6.9–20.7 (one-sided *p* = 0.047), indicating that the primary endpoint was met. The CR rate in the persistent phase (120–504 h) was also higher in the olanzapine group than in the placebo group (64.9% vs. 44.4%, difference 19.4%, 95% CI 2.4–35.3). The daily CR rate and daily no-nausea rate were higher in the olanzapine group than in the placebo group throughout the 21-day observational period. The adverse events were similar to those previously reported for olanzapine, and no new safety signals were identified in either group. The addition of olanzapine appeared to be effective in preventing NV in patients receiving T-DXd. The results revealed that patients who underwent T-DXd treatment experienced persistent NV during the 21-day cycle. However, the study left unresolved questions regarding the use of olanzapine vs. NK1RA, as well as the optimal dosage of olanzapine and duration of treatment.

**Table 3:** Clinical trials evaluating prophylactic antiemetic therapy in patients receiving T-DXd

Study name Phase Trial number	Cancer type	*N*	Method	Results
Ooki, et al EN-hance study [24] Phase II jRCTs031200336	HER2-positive gastric cancer	60	aprepitant, palonosetron, and DEX vs. palonosetron and DEX	CR rates (0–504 h): 37.3% vs. 41.4%
Iihara, et al. [25] Phase II UMIN000041004	HER2-positive metastatic breast cancer	40	Aprepitant/fosaprepitant, granisetron, and DEX vs. granisetron and DEX	CR rates (0–120 h): 70.0% vs. 36.8%
Sakai, et al ERICA study [26] Phase II jRCTs031210410	HER2-positive and HER2-low metastatic breast cancer	168	Olanzapine, palonosetron/granisetron, and DEX vs. placebo, palonosetron/granisetron, and DEX	CR rates (24–120 h): 70.0% vs. 56.1%

*DEX* dexamethasone, *vs.* versus, *CR* complete response

## Sacituzumab govitecan

Sacituzumab govitecan (SG) is composed of a humanized trophoblast cell surface antigen 2 (TROP2)-directed monoclonal antibody conjugated to a topoisomerase I inhibitor drug (SN-38) via a hydrolysable linker [34]. SN-38 is an activated form of irinotecan, and the DAR of SG is 7.6 [35]. The frequencies of NV in patients receiving SG were approximately 55% and 20%, and the frequencies of grade ≥ 3 NV were low (Table 4) [4, 36]. The median time to first-onset NV was 8 and 24.5 days after the initiation of SG treatment in the ASCENT study [37]. The protocol of ASCENT recommends prophylactic antiemetic therapy with 5-HT3 RA [4]. However, premedication or concomitant medication for NV was reported to be used in 86% of the SG group [37]. The TROPiCS-02 protocol states that sacituzumab govitecan is considered moderately emetogenic, and prophylactic 2-drug antiemetic therapy is recommended. Moreover, if NVs are persistent, a 3-drug regimen may be used, including 5-HT3 RA, an NK1RA, and dexamethasone (10 mg PO or IV) [36].

**Table 4:** Incidence and severity of NV among patients who received sacituzumab govitecan in clinical studies

Study name Phase	Cancer type	*N*^a^	Dose of SG	Nausea (%)	Vomiting (%)
ASCENT Phase III [4]	Triple negative, metastatic breast cancer	258	10 mg/kg, day 1 triweekly	Any grade: 57 Grade ≥ 3: 3	Any grade: 29 Grade ≥ 3: 1
TROPiCS-02 Phase III [36]	HR-positive, HER2-negative, metastatic breast cancer	268	10 mg/kg, day 1 triweekly	Any grade: 55 Grade ≥ 3: 1	Any grade: 19 Grade ≥ 3: < 1
TROPHY-U-01 Phase II, cohort 1 [39]	metastatic urothelial carcinoma	113	10 mg/kg, day 1 triweekly	Any grade: 60 Grade3: 4 Following grades: 0	Any grade: 30 Grade3: 1 Following grades: 0

^a^ Number of patients who received SG and were evaluable for safety

*NV* nausea and vomiting, *HR* hormone receptor, *SG* Sacituzumab govitecan

The emetogenic risk of SG and the recommended antiemetic therapy are also inconsistent across clinical guidelines (Table 5). The MASCC and ESMO guidelines classify SGs at the high end of the moderate-risk category, such as carboplatin [15]. The NCCN classifies SGs as high risk [14]. The emetic risk of SG is not still defined by ASCO guidelines [16]. The JSCO guidelines also classify SG as the high end of the moderate-risk category [17].

**Table 5:** Emetic risk and recommended antiemetic therapy in recent guidelines

Guidelines	Last updated	Emetic risk of SG	Recommended antiemetic therapy
MASCC and ESMO [15]	2023	High end of the moderate-risk category, most closely resembling that of carboplatin	NK_1_-RA, 5-HT3 RA, and DEX^a^
NCCN [14]	2024	High	NK_1_-RA, 5-HT3 RA, DEX, and OLZ 5-HT3 RA, DEX, and OLZ NK_1_-RA, 5-HT3 RA, DEX
ASCO [16]	2020	–	–
JSCO [17]	2023	High end of the moderate-risk category	5-HT3 RA, and DEX^b^

^a^ The recommended regimen for carboplatin with an AUC ≥ 5

^b^ The guidelines state that there is potential for SG to be considered a candidate for combination with NK_1_-RA-based triplet therapy

*MASCC* Multinational Association of Supportive Care in Cancer, *ESMO* European Society for Medical Oncology, *NCCN* National Comprehensive Cancer Network, *JSCO* Japan Society of Clinical Oncology, *T-DXd* trastuzumab deruxtecan, *NK1-RA* neurokinin 1 receptor antagonist, *5-HT3 RA* serotonin type 3 receptor antagonist, *DEX* dexamethasone

No prospective clinical study evaluating optimal prophylactic antiemetic therapy in patients receiving SG has been reported. Treatment with SG is scheduled for days 1 and 8 of a 21-day cycle, and given that NV tends to occur later (the median time to onset of nausea was 8 days and that of vomiting was 24.5 days in the ASCENT), it is necessary to develop an antiemetic regimen tailored to the onset of these symptoms [37]. There is no evidence indicating the efficacy of weekly administration of NK1RA. A previous randomized clinical trial revealed no difference between weekly and triweekly fosaprepitant in terms of the primary outcome of CR in patients receiving concurrent chemoradiotherapy [38].

## Datopotamab deruxtecan

Datopotamab deruxtecan (Dato-DXd) is a TROP2-directed ADC with a potent Topo I inhibitor, DXd. T-DXd and Dato-DXd adopt the same linker system and payload, and Dato-DXd has a lower DAR of 4 compared to 8 for T-DXd [40]. The incidence of NV is lower than that with T-DXd, likely due to a lower DAR [2, 41]. Although the emetic risk of Dato-DXd is still not defined by the NCCN guidelines, MASCC/ESMO guidelines, or ASCO, it seems to be a moderate emetic risk considering the incidence of vomiting in pivotal studies (Table 6). The protocol of the TROPION-Breast01 study recommended that subjects receive prophylactic antiemetic therapy such as 5-HT3 RA or NK1 RA and/or steroids [41].

**Table 6:** Incidence and severity of NV among patients receiving Datopotamab deruxtecan in clinical studies

Study name Phase	Cancer type	*N*^a^	Dose of Dato-DXd	Nausea (%)	Vomiting (%)
TROPION-PanTumor01 Phase I [42]	HR + /HER2 − metastatic breast cancer	41	6 mg/kg day 1 triweekly (N = 83) 8mg/kg day1 Triweekly (N = 2, both are triple-negative type)	Any grade: 56 Grade ≥ 3: 0	Any grade: 24 Grade ≥ 3: 0
	Triple negative metastatic breast cancer	44		Any grade: 66 Grade ≥ 3: 2	Any grade: 39 Grade ≥ 3: 5
TROPION-PanTumor01 Phase I [43]	NSCLC	50	4 mg/kg day 1 triweekly	Any grade: 48 Grade ≥ 3: 0	Any grade: 14 Grade ≥ 3: 0
		50	6 mg/kg day 1 triweekly	Any grade: 64 Grade ≥ 3: 4	Any grade: 18 Grade ≥ 3: 2
		80	8 mg/kg day 1 triweekly	Any grade: 54 Grade ≥ 3: 1	Any grade: 36 Grade ≥ 3: 0
TROPION-Breast01 Phase III [5]	HR + /HER2 − metastatic breast cancer	360	6 mg/kg day 1 triweekly	Any grade: 51 Grade ≥ 3: 1	Any grade: 20 Grade ≥ 3: 1

^a^ Number of patients who received Dato-DXd and were evaluable for safety

*NV* nausea and vomiting, *HR* hormone receptor, *Dato-DXd* datopotamab deruxtecan

## Discussion

ADCs represent a novel class of targeted cancer therapeutics. We believe that there are two major future challenges in managing NV induced by ADCs. First, the development of personalized antiemetic therapy that is tailored to the onset and duration of NV and to patient risk is anticipated. This approach could enhance efficacy and reduce financial toxicity. The ERICA study revealed that the degree and duration of NV induced by T-DXd varied among patients. Future studies to identify prognostic and predictive factors in a larger cohort are anticipated. Who are the most likely to benefit from using olanzapine, NK1RA, or the combination of both drugs for prophylaxis? These questions should be answered to through future clinical trials. Personalized biomarker-based approaches are also expected to be developed. There are data suggesting that certain cytokines and peptides could be potential biomarkers for CINV [44, 45]. Second, decentralizing supportive care is crucial, as more patients seek care outside traditional hospital settings. The persistent NV induced by T-DXd and the delayed onset of NV induced by SG highlight the importance of decentralizing supportive care. Advances in telemedicine and remote monitoring now allow patients to manage side effects such as nausea from home, reducing the need for hospital visits. The utility of monitoring using ePRO, which triggers alerts during symptom exacerbation, has been demonstrated in several clinical trials and is expected to be widely implemented in future trials of supportive care [46]. A possible study design could be employed in clinical trials that adjusts antiemetic regimens for the following cycles based on the symptom during the first cycle. Addressing these challenges will require collaboration among healthcare providers, researchers, patients and technologists.

In conclusion, further studies are warranted to establish more patient-centered supportive care for ADC-induced NV.
